# Draft Genome Sequence of the Antitrypanosomally Active Sponge-Associated Bacterium *Actinokineospora* sp. Strain EG49

**DOI:** 10.1128/genomeA.00160-14

**Published:** 2014-03-06

**Authors:** Janno Harjes, Taewoo Ryu, Usama Ramadan Abdelmohsen, Lucas Moitinho-Silva, Hannes Horn, Timothy Ravasi, Ute Hentschel

**Affiliations:** a Department of Botany II, Julius-von-Sachs Institute for Biological Sciences, University of Würzburg, Würzburg, Germany; b Division of Chemical and Life Sciences and Engineering and Division of Applied Mathematics and Computer Science, King Abdullah University of Science and Technology, Thuwal, Saudi Arabia

## Abstract

The marine sponge-associated bacterium *Actinokineospora* sp. strain EG49 produces the antitrypanosomal angucycline-like compound actinosporin A. The draft genome of *Actinokineospora* sp. EG49 has a size of 7.5 megabases and a GC content of 72.8% and contains 6,629 protein-coding sequences (CDS). antiSMASH predicted 996 genes residing in 36 secondary metabolite gene clusters.

## GENOME ANNOUNCEMENT

The bacterial strain *Actinokineospora* sp. EG49 was isolated from the inner tissues of the marine sponge *Spheciospongia vagabunda*, which was collected from the Red Sea (1). The genus *Actinokineospora* belongs to the family *Actinosynnemataceae* under the phylum *Actinobacteria* (2). Members of *Actinokineospora* are widespread in terrestrial environments, including soil and plants (3, 4), and were recently isolated from marine sponges (1). So far only two draft genomes of the genus *Actinokineospora* are available (BioProject identification [ID] 188797 and 174972 [http://www.ncbi.nlm.nih.gov/]). Two novel angucycline-like compounds named actinosporin A and B were isolated from *Actinokineospora* sp. EG49, of which actinosporin A displayed antiparasitic activity against *Trypanosoma brucei brucei* (5). Besides chemical characterization, we pursued genomic mining of *Actinokineospora* sp. EG49 in order to discover new bioactive compounds through the identification and characterization of cryptic biosynthetic gene clusters.

The genomic DNA of *Actinokineospora* sp. EG49 was sequenced using the Illumina HiSeq 2000 system. The draft genome was obtained from a total of 199,066,576 read pairs that were produced with a mean read length of 101 base pairs (bp), representing a mean coverage of 1,616-fold. Reads were assembled *de novo* using AbySS (6) with a k-mer size of 55, resulting in 263 contigs of >200 bp in size and an *N*_50_ value of 52,481. Considering all contigs, the draft genome size is 7,529,476 bp with a GC content of 72.8%, which compares to the genomes of *Actinokineospora enzanensis* DSM 44649 (8,119,858 bp, GC content 70.8%) (http://www.ncbi.nlm.nih.gov/assembly/GCA_000374445.1/) and *Actinokineospora inagensis* DSM 44258 (7,278,759 bp, GC content 70.2%) (http://www.ncbi.nlm.nih.gov/assembly/GCA_000482865.1/). The gene annotation was performed using RAST (7), tRNAscan-SE (8), and RNAmmer (9), yielding a total of 6,629 coding sequences (CDS) and 50 tRNA and 3 rRNA genes.

By use of the online platform for genomic mining antiSMASH (10), a total of 996 genes residing in 36 gene clusters for putative biosynthetic secondary metabolites were predicted. These divide into genes for five type I polyketide synthases (PKSI), four PKSII, one PKSIII, and four nonribosomal peptide synthetases (NRPS), three gene clusters involved in terpene biosynthesis, three gene clusters related to bacteriocin biosynthesis, and genes for five hybrid NRPS-PKSI. In addition, one gene cluster each for hybrid NRPS-PKSII, hybrid NRPS-beta-lactam, hybrid NRPS-terpenes, hybrid NRPS-ectoine, ectoine, lantipeptide, and siderophore and four other gene clusters were predicted. Furthermore, the secondary metabolite gene analysis tool NaPDoS (11) predicted the presence of natural products such as actinorhodin, alnumycin, saquayamycin, and tetronomycin, based on a search for polyketide ketosynthase domains. Alnumycin and saquayamycin are structurally similar to actinosporins, and both are produced by a type II polyketide synthase (5). Genes coding for a ketosynthase involved in the first steps of fatty acid biosynthesis with reported homologues that are active in polyketide biosynthesis were also predicted by NaPDoS (11). These predictions indicate the genomic potential of one isolate of the rare genus *Actinokineospora* for the production of diverse natural products.

### Nucleotide sequence accession numbers.

The draft genome sequence of *Actinokineospora* sp. strain EG49 was submitted to DDBJ/EMBL/GenBank as a whole-genome shotgun project under the accession number AYXG00000000. The version described in this paper is the first version, AYXG01000000.
